# Supplementary material for: Post-Intensification Poaceae Cropping: Declining Soil, Unfilled Grain Potential, Time to Act
Source: Plants (Basel). 2023 Jul 24;12(14):2742. doi: 10.3390/plants12142742 (PMC10384148; doi:10.3390/plants12142742)
Supplement: Supplementary file 1 [file plants-12-02742-s001.zip › plants-2484529-supplementary.pdf]

Supplementary File: Cropping systems, agronomic inputs, fields, grain.

Squire, G.R.; Young, M.W.; Banks, G. 2023. Supplementary materials on cropping systems and agronomy and photographs of field sites and grain.

The various cropping systems in the region are compared in Table S1 in terms of the timing of tillage and sowing, crops, source and indicative amount of nitrogen fertiliser and degree and period of pest control. Table S2 gives agronomic inputs and yield for the main crops studied in this paper, spring barley and winter wheat, summarised from government surveys for the 2014 harvest year.

Photographs are given in Plates S1 to S3 of various aspects of the study. Plate S1 shows four fields sampled in the region in 2014. Nitrogen application determines early canopy growth and ultimately dry matter production, yield and C acquisition. Plate S2 shows examples of three quadrats (side length 0.5 m) in crops receiving high, medium and low applications of nitrogen. Plate S3 shows photographs of grain from barley and wheat.

Table S1. General characteristics of cropping systems adapted from previous summaries for this region [64]. N-fertiliser varies between crops in each system, indicative ranges given; (z) indicates zero mineral fertiliser and zero chemical pesticide in some farming systems. W indicates long-season winter and S short-season spring varieties.

System	Tillage and sowing	Main crops	Nitrogen fertiliser	Pest control
Spring crops and grass	in spring, over-winter stubble in crops, no tillage in 2-3 year grass ley	S barley, S oats, sown grass ley	livestock manure and mineral, 50-100 kg ha^{-1} (z)	none to grass, chemical control in most crops, period 6-7 months annually (z)
Mainly spring crops	spring, over-winter stubble	S barley, S oats, oilseed rape	mineral or mineral with livestock manure, 80-120 kg ha^{-1} (z)	chemical control to most crops, period 6-7 months annually (z)
Mixed spring and winter crops	autumn and spring in different years	W wheat, W barley, S barley, S oats, oilseed rape	mineral or mineral with livestock manure, 100-200 kg ha^{-1} (z)	chemical control to most crops, period 6-11 months annually (z)
Mainly winter crops	mainly autumn	W wheat, W barley, oilseed rape, potato	mineral 150-220 kg ha^{-1}	high chemical control in all crops, period 11 months, >10 treatments per year

Table S2. Spring barley and winter wheat agronomic data from government survey in Scotland in the year of sampling (2014). Average fertiliser applied in livestock manures is not available (na) in comparable form from standard survey.

Agronomy/operations	Spring barley	Winter wheat
Sowing period	March, April	September, October
Harvest period	August	August
Fertiliser [64]		
% crop area treated, mean application in kg ha^{-1}:		
Nitrogen	99%, 107	100%, 179
Phosphorus (P_2O_5, kg/ha)	94%, 56	70%, 62
Potassium (K_2O, kg /ha	92%, 72	76%, 85
Livestock manure	36%, na	15%, na
Pesticide [69]		
% crop area treated, treatments per season:		
Herbicide	95%, 2-3	95%, 2-3
Fungicide	91%, 2-3	100%, 7-8
Insecticide	17%, 1	42%, 1
Growth regulators	25%, 1	98%, 2-3
Seed treatment	88%, 1	95%, 1
Crop [65]		
Mean yield, t ha^{-1}	6.07	9.07
Percentage of cereal area	59%	24%

Plate S1. Examples of landscapes and sampled fields at different stages of crop development.

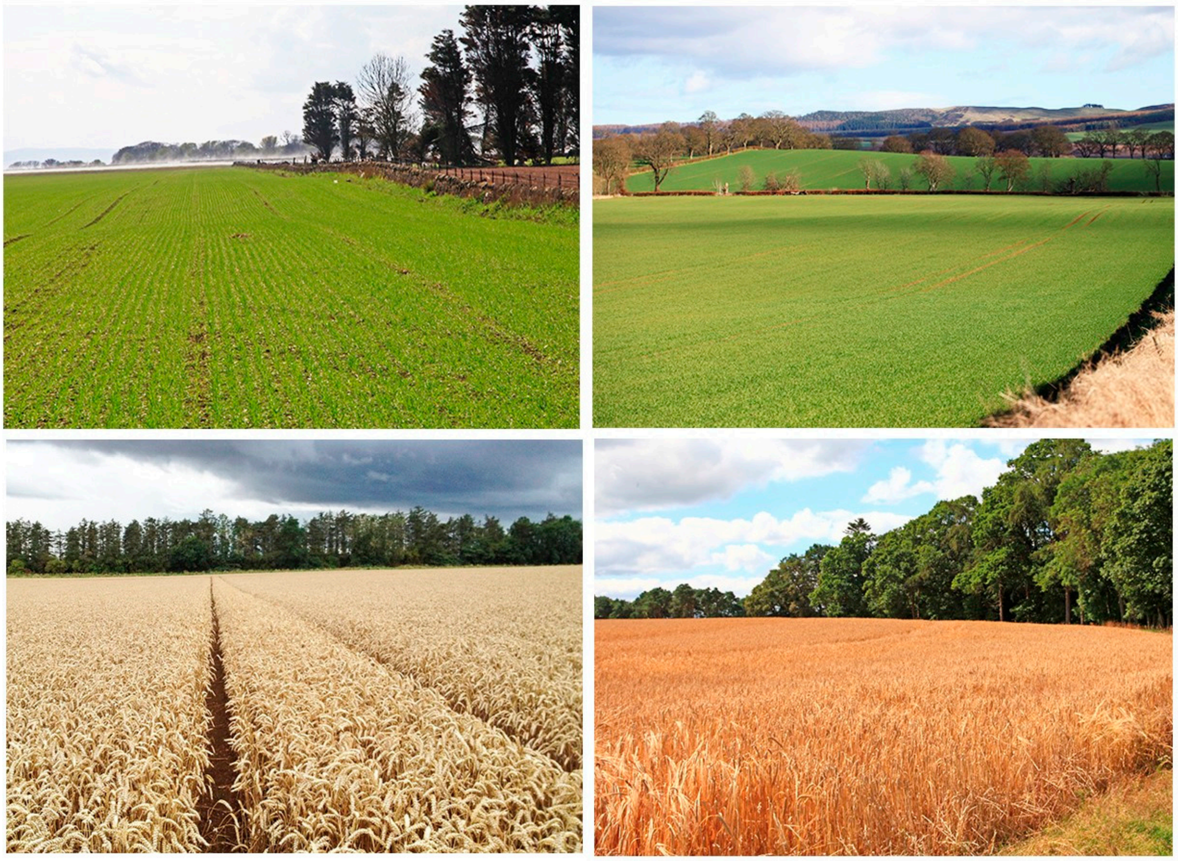

Examples of fields sampled: (upper) in spring; and (lower) just before harvest (left wheat, right barley).

Plate S2. Examples to show the range of crop leaf cover for wheat crops in April. Canopy growth at this time and subsequently is determined mainly by applied nitrogen fertiliser. The examples indicate the range low to high N that determines the values of C-crop in Fig. 3 and head dry mass and grain number in Fig. 6. Weeds were absent due to chemical control in most high-input crops but emerged and coexisted with low-input crops (right).

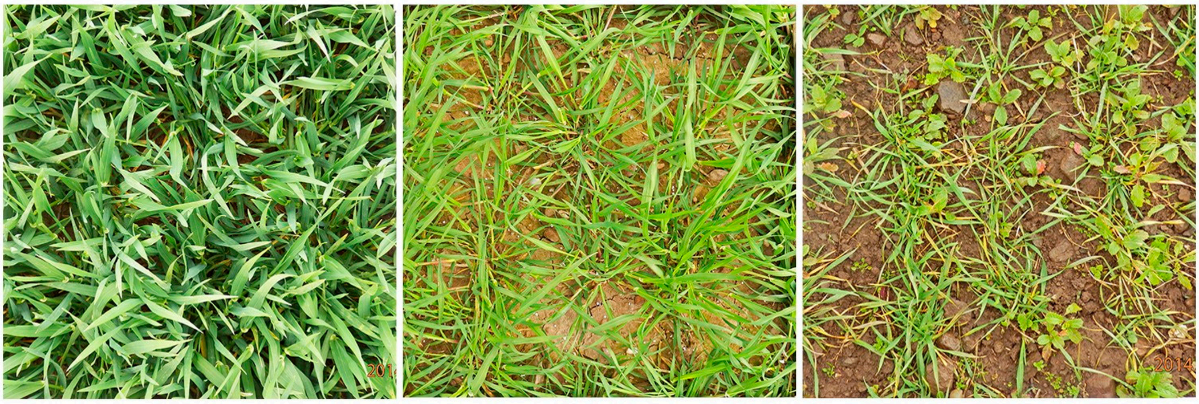

Examples of wheat crops in spring: left to right, decreasing crop cover due to reduced N inputs, right showing lowest crop cover with weeds, side length of square 0.5 m.

Plate S3. Images of grains at the same magnification to compare barley (left) and wheat right.

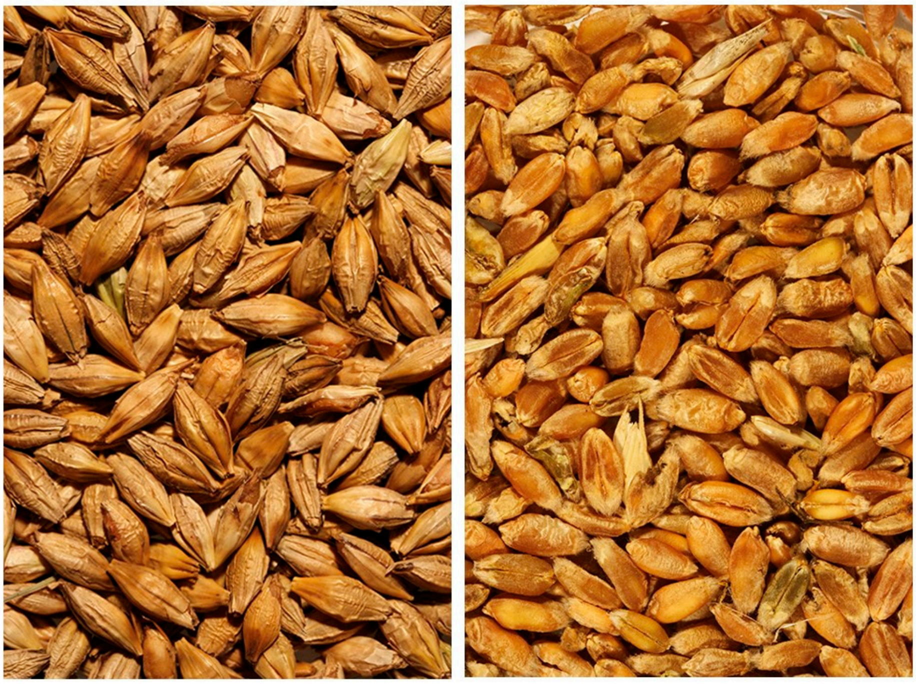

Grains of barley (left) and wheat (right), each 6-8 mm in length.
